# A simple cognitive method to improve the prediction of matters of taste by exploiting the within-person wisdom-of-crowd effect

**DOI:** 10.1038/s41598-022-16584-7

**Published:** 2022-07-20

**Authors:** Itsuki Fujisaki, Hidehito Honda, Kazuhiro Ueda

**Affiliations:** 1 Graduate School of Arts and Sciences, The University of Tokyo, Tokyo, Japan; 2 Faculty of Psychology, Otemon Gakuin University, Osaka, Japan

**Keywords:** Psychology, Human behaviour

## Abstract

In our daily lives, we must often predict the level of others’ satisfaction with something they have not experienced thus far. How can such a prediction be accurate? Existing studies indicate that, by referring to the extent to which people themselves have enjoyed something, they are able to predict others’ future satisfaction, to some extent. In this study, we propose a method that can further improve such predictions. This method is expected to allow individuals to exploit the ‘wisdom of the crowd’ within a person, in terms of taste. Specifically, for a single target, participants in our study group produced two opinions from different perspectives: the degree to which they preferred something, and they estimated ‘public opinion’. Utilising two behavioural studies and computer simulations, we confirmed the effectiveness of our method; specifically, blending the two opinions could enhance an individual’s prediction ability. Subsequently, we mathematically analysed how effective our method is and identified several factors that influenced its efficiency. Our findings offer several contributions to ‘wisdom-of-crowd’ research.

## Introduction

In daily life, we are often asked questions such as, ‘Do you feel I will like the restaurant you visited?’ or ‘Do you think I will be satisfied with the lecture you attended last year?’ by our friends or peers. How can we accurately predict others’ future satisfaction in such situations^1–4^? An intuitive way is to speculate on how much others would enjoy their experiences. However, existing studies^5–7^ noted that these speculations tend to be inaccurate, even when one has a long relationship with them. In contrast, some researchers^7–9^ have revealed that our own impression can be a good predictor. Specifically, simply answering the extent to which we ourselves appreciated the experience (hereinafter referred to as ‘Own’ opinion) can predict others’ future satisfaction, to some extent.

This study proposed a method that can further improve these predictions. Our proposed method is as follows: for a question item (e.g. ‘Do you think I would like the restaurant?’), two opinions should be considered, from two different perspectives: in addition to the Own opinion, they must estimate the public’s views (hereinafter called the ‘Estimated’ opinion). Specifically, they would guess the extent to which average people prefer it. Subsequently, in our study, the two opinions are then averaged (hereinafter referred to as ‘Blended’ opinion). This study’s central hypothesis is that the Blended opinion is a better predictor than the Own opinion.

Its theoretical basis can be explained as follows. We developed a method based primarily on wisdom-of-crowd research^10–18^. It has been established that the aggregate of multiple judgements could have greater accuracy than individual ones. Note, however, that judgement problems are generally matters of *fact*, in which there is an objective truth (e.g. ‘What percentage of the world’s airports are in the United States?’). Importantly, some previous studies^2,3,19^ show that the wisdom-of-crowd effect can also emerge for matters of *taste,* in which there is no objective, universal truth. Put simply, these studies show that, as the number of people voicing their opinion increases, the accuracy of the prediction (i.e. the aggregation of their opinions) also increases. Furthermore, recent studies^4,26–35^ demonstrate that an individual can harness the within-person wisdom of the crowd (called ‘the wisdom of the inner crowd’). In these studies, an individual produced multiple responses for a problem (basically regarding the matters of *fact*, see ‘Discussion’), resulting in the aggregated answer being more accurate than a single one. Overall, we consider that an individual can improve their prediction if they can harness the within-person wisdom of the crowd for matters of taste.

As mentioned above, participants in our study were requested to produce two evaluations for the same item: the Own and the Estimated opinion. To obtain the Estimated opinion, participants had to examine the item from a different perspective than their own opinion; in other words, our method was expected to produce two quasi opinions from participants. This approach for obtaining ‘the wisdom of the inner crowd’ is in line with the methods of previous studies^4,20–29^. To determine the details of the Estimated opinion—the simulated public opinion—we draw on findings from cognitive and social psychology. In these fields, many studies have examined how people can think differently from their own. In particular, it is well-known that, to consider others’ perspectives, an individual can make diverse recognitions in various aspects, called ‘perspective-taking’^30^. For example, perspective-taking enables a person to decrease stereotypic biases^31^, engage in coordinated behaviours^32^, change preferential values^33^, and reduce egocentric thinking^34^. We therefore decided to primarily follow the perspective-taking paradigm. Subsequently, among ‘others’, we adopted the general crowd’s viewpoint. Many studies have reported that people believe that the general crowd differs from themselves in several ways (e.g. the degree of intelligence^35–38^, risk attitude^39,40^, and judgemental estimations^26^). Accordingly, we hypothesised that, by considering the general crowd’s perspectives, participants could make evaluations that differ from their own opinions for the same target.

This is a broad background of our method. In the following section, we report on our comprehensive investigation on the effectiveness of the proposed method. We first conducted two experimental studies to collect the evaluation data (see details in ‘Methods’) that differed in terms of the stimuli category presented: paintings and music in Studies 1 and 2, respectively. Both studies evaluated stimuli successively using our method. Subsequently, we utilised the evaluation data and conducted a computer simulation to assess the effectiveness of our method.

## Results

### Analysis

**Figure 1:**
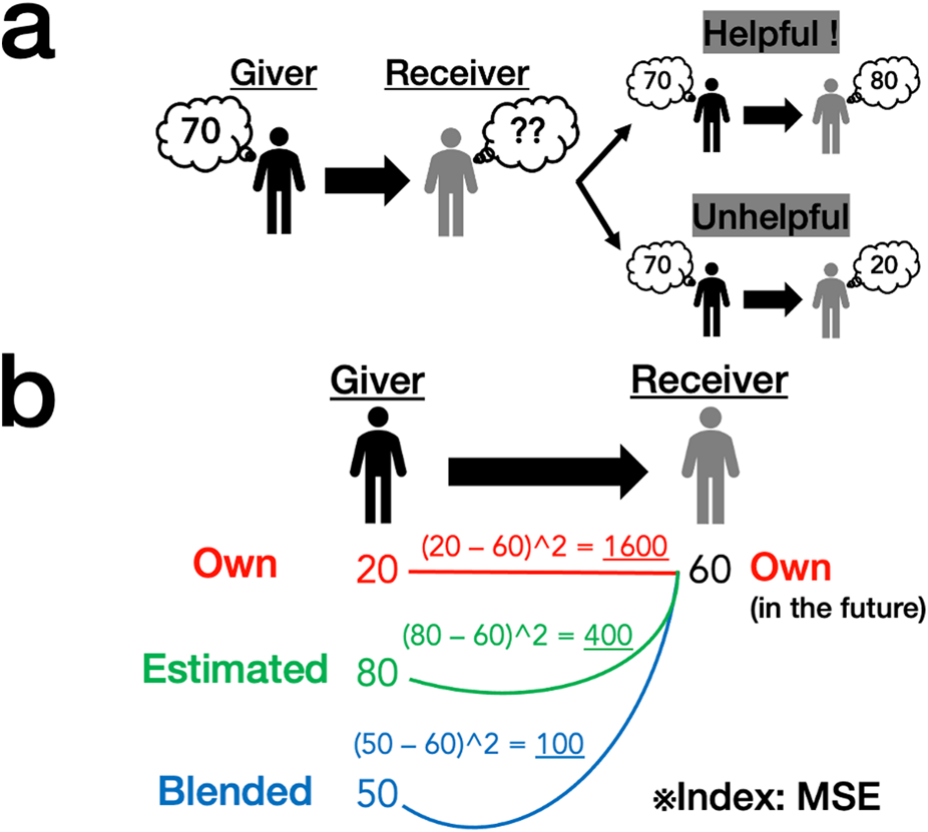
Illustration of the analysis. (**a**) Definition of the helpfulness of a Giver’s opinion. When the Giver’s opinion can accurately predict a Receiver’s (future) preference (the right upper column), we define the opinion as ‘helpful’ for the Receiver. Conversely, when the Giver’s opinion inaccurately determines the Receiver’s future choice (the right lower column), we consider the opinion as ‘not helpful (unhelpful)’. (**b**) Computation of the efficacy (helpfulness) of a Giver’s opinion. We set the Giver’s Own, Estimated, and Blended opinions, after which we calculated the MSE (Mean Squared Error) between each of the Giver’s opinions and a Receiver’s future satisfaction (the Receiver’s Own opinion). A smaller MSE indicates that the Giver’s opinion is more helpful. Through the analysis, all participants except the Giver became Receivers. Additionally, we computed the MSE for all participants including the Giver.

In the analysis, we focused on the following situations: For a target, one person (‘Giver’) gave an opinion to another (‘Receiver’). Figure 1a illustrates the definition of ‘helpfulness’ of the Giver’s opinion. The left side represents the following situation: the Giver has already experienced a target; then, they give an opinion (for example, 70 in the figure) to a Receiver, who has not experienced it thus far. The right side shows the results of the opinion giving. Here, the Receiver has also encountered the target and has formed their own judgement (that is, the Receiver’s Own opinion). The upper row on the right side demonstrates the situations in which the Giver had a similar opinion (80) regarding the target. In this case, we assumed that the Giver’s opinion was ‘helpful’, as it accurately predicted the Receiver’s future satisfaction. Conversely, when the Receiver had an opinion different from the Giver (for example, 20 in the lower row), we supposed that the Giver’s opinion was relatively ‘unhelpful’.

Note that this assumption is similar to those adopted in previous studies^3,4,7,8,19^. As mentioned in the Introduction section, we simulated the opinion giving on a computer using the evaluation data in Studies 1 and 2. The simulations eliminated the possibility that the Receiver’s opinion formation was influenced by receiving the Giver’s opinion.

For the detailed analysis, we mainly used the theoretical framework of existing wisdom-of-crowd literature on matters of taste (particularly, Müller-Trede et al.^3^), which enabled us to quantitatively investigate the efficacy of the proposed method.

Figure 1b illustrates the detailed analysis. A Giver and a Receiver were independently selected from the participants whose behavioural data were obtained, after which we examined the helpfulness of the Giver’s three opinions (Own, Estimated, and Blended opinions). As shown in Fig. 1b, we employed the mean squared error (MSE) as an index for the helpfulness of opinions. In particular, we computed the value of the squared difference between a Giver’s opinion and the Receiver’s Own opinion. A smaller MSE value indicated that the Giver’s opinion was more helpful. We conducted this analysis across all stimuli.

Using the simulation procedure, we examined all the Giver–Receiver combinations: (i) For a Giver, all participants except the Giver became their receivers, and we computed the MSE; (ii) The average MSE across all Receivers was allocated to the Giver’s value; (iii) We computed the MSE for all participants including the Giver.

### Main results

**Figure 2:**
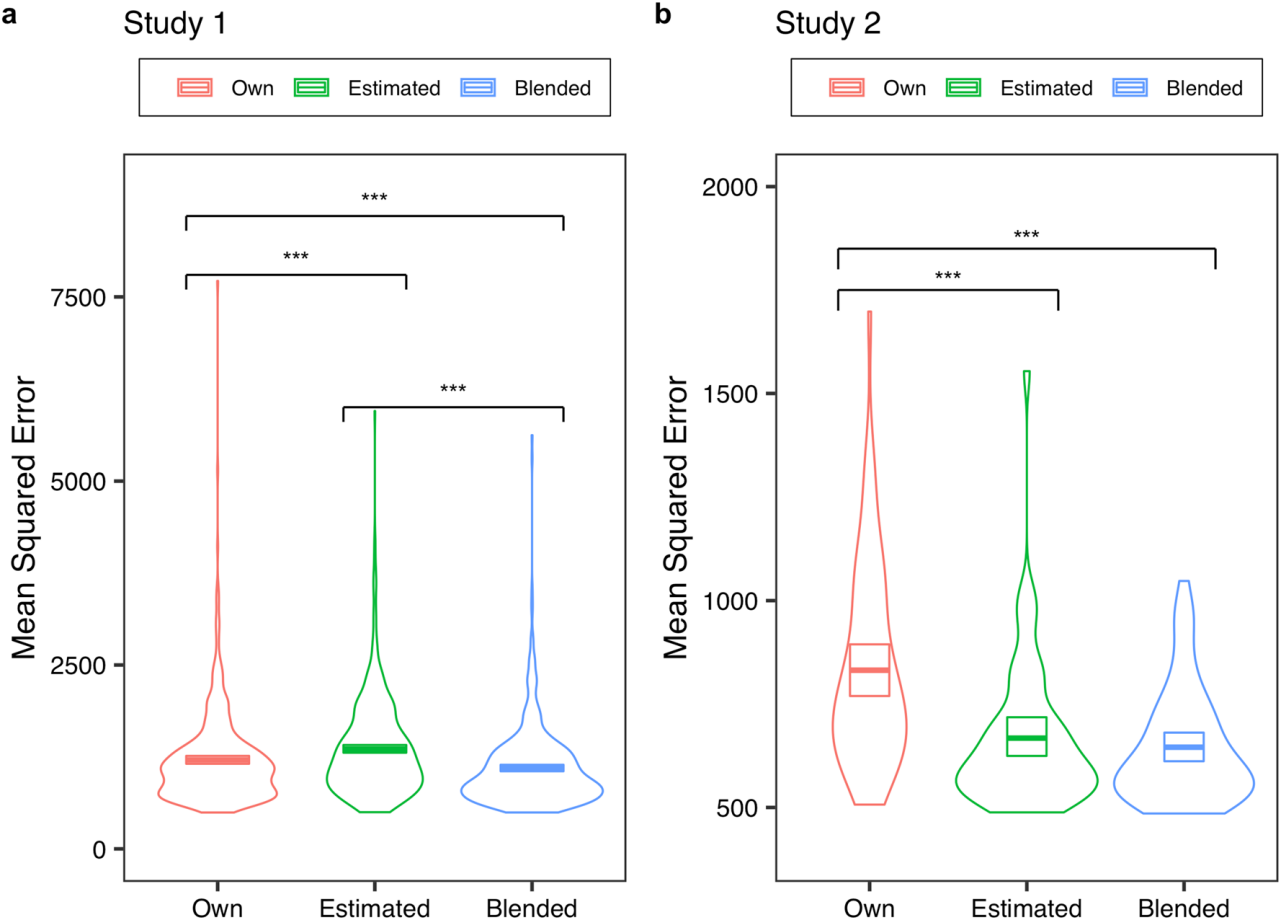
Results of the main analysis. The figures show the MSE (Mean Squared Error) for each Giver in a violin plot with a boxplot. Note that all boxplots indicate 95% confidence intervals for convenience. In this study, we conducted all bootstrapping based on a sample of 1,000 with replacement. As hypothesised, the Blended opinion recorded a lower MSE than the Own opinion (*ps* < .001).

Figure 2 shows the results of the analysis. Notably, we obtained a lower MSE for the Blended opinion than the Own opinion across the two studies (Study 1: *Wilcoxon signed-rank* test*, p* < 0.001, Cliff’s *delta* = 0.37; Study 2: paired *t*-test, *p* < 0.001, Cohen’s *d* = 1.36). That is, using our method, a Giver could improve the accuracy of their opinions. Thus, our main hypothesis was supported.

**Figure 3:**
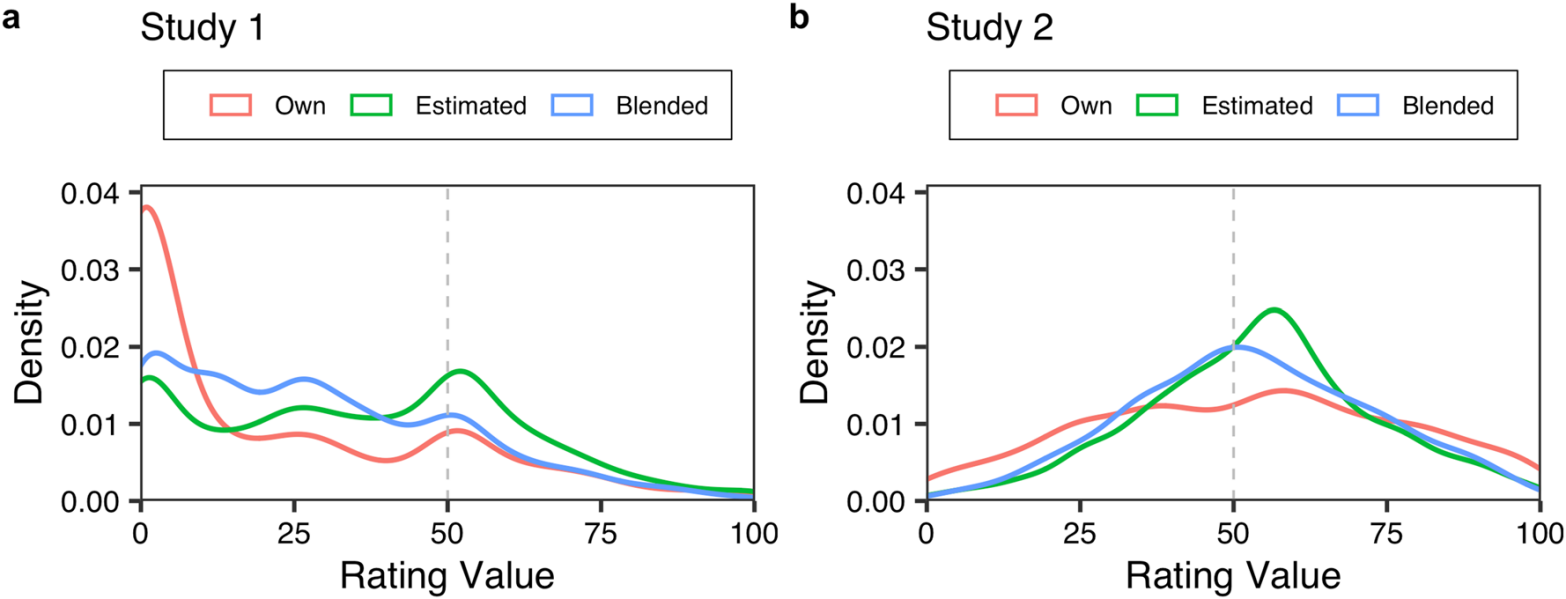
Data structure in Study 1(**a**) and Study 2(**b**). Each line represents a probability density function. In Study 1, not all opinion types followed a normal distribution (*Kolmogorov–Smirnov* test: *ps* < 0.001), while in Study 2, they did (*ps* > 0.1).

As mentioned in the Introduction section, the two studies differed in terms of stimulus categories. The results indicated that they also differed in terms of the data structure, as shown in Table 1. In Study 1, the average rating values were less than half (i.e. 50) across all opinions. However, in Study 2, these values hovered around half. Additionally, as shown in Fig. 3, none of the opinions followed a normal distribution in Study 1, while in Study 2, they did (*Kolmogorov–Smirnov* test; Study 1: *ps* < 0.001; Study 2: *ps* > 0.1). Taken together, our method was effective across different categories and data structures.

**Table 1:** Data structure across the two studies. This table shows a 95% confidence interval (CI) regarding the rating values. We computed the 95% CI by bootstrapping. In Study 1, the average rating values for all types of opinions were below half, while in Study 2, they were around half.

	Study 1	Study 2
Own	[21.05, 22.95]	[50.75, 53.53]
Estimated	[35.00, 36.88]	[53.00, 55.05]
Blended	[28.07, 29.83]	[51.99, 54.08]

**Figure 4:**
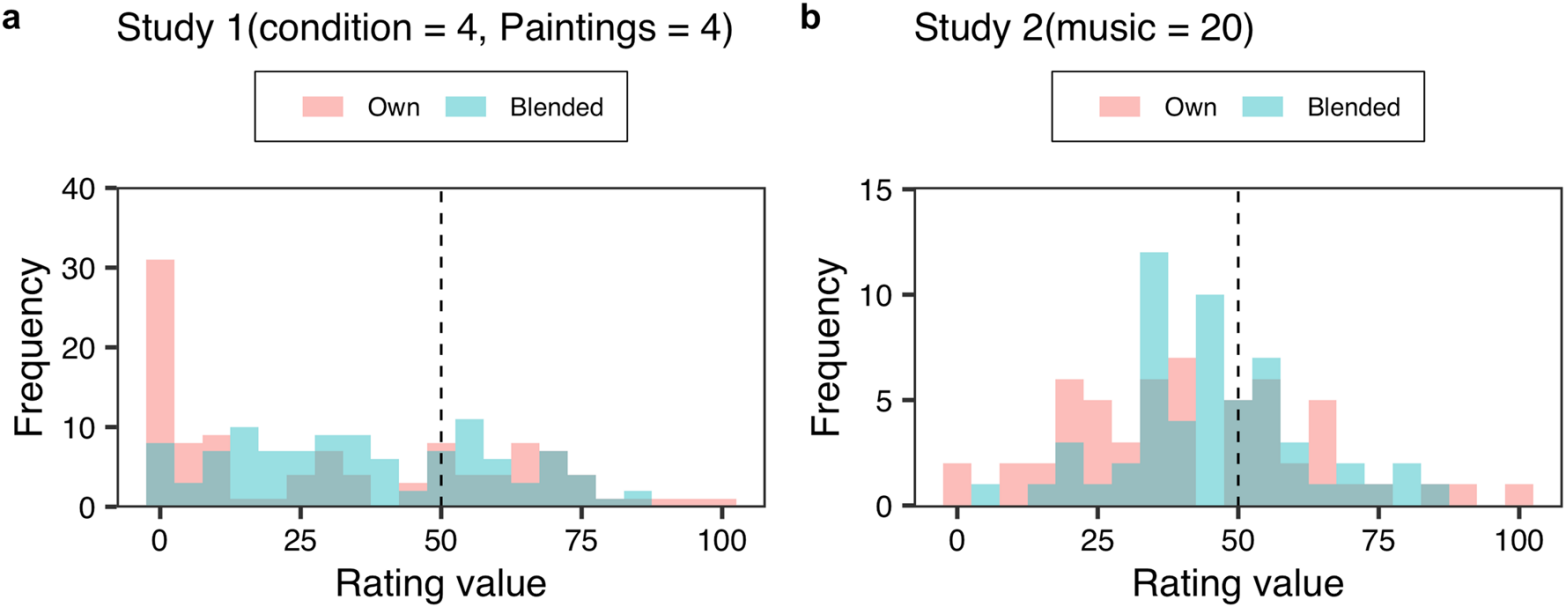
Typical example of the results in Study 1(**a**) and Study 2(**b**). Each histogram indicates the number of participants (Frequency). The titles represent the stimulus numbers (see Supplementary Tables S1 and S2).

Next, we discuss how effective our method was across different data structures. Figure 4 represents typical examples of the results. In Study 1 (Fig. 4a), the rating values of Own opinion focused on 0. However, there were a few large rating values (for example, 100). In these cases, the MSE of Own became quite large. Conversely, fewer Blended ratings were 0. Specifically, the rating value of Blended opinion tended to remain distributed between 0 and 75. This resulted in Blended recording a lower MSE than Own. Subsequently, in Study 2 (Fig. 4b), the mean rating value of Own was similar to that of Blended (especially around 50). However, the distribution of the rating values of Own was relatively larger than that of Blended. That is, there were certain cases where the rating values between a Giver and a Receiver were largely different (for example, the Giver’s rating value was 0 and the Receiver’s rating value 100, and vice versa). In this respect, there were relatively fewer cases in the Blended than in the Own opinion, as the Blended distribution was small. Thus, our method was effective across different data structures.

Notably, the results also indicated that Blended had a significantly lower MSE than Estimated in Study 1 (*p* < 0.001; Cliff’s *delta* = 0.52). Although we did not find such an effect in Study 2 (*p* = 0.55, Cohen’s *d* = 0.18), the findings were also in favour of Blended; we calculated the number of participants who had a lower MSE in Estimated (Blended), compared to Own opinion. We found that in Blended, more participants had a lower MSE than in Estimated (55 for Blended and 44 for Estimated, out of 56 participants; *Fisher’s exact* test: *p* < 0.005). When we consider these results along with those of Study 1, it can be stated that Blended opinion improves the Giver’s opinion more effectively than Estimated opinion.

### How our method performed: analysis by decomposing MSE

How could our method reduce the error in Own opinion? It is well-known^3,41,42^ that the MSE was theoretically decomposed into different sources of error. By performing this decomposition, we were able to comprehensively observe how effective our method was. Several decompositions of the MSE have been suggested in the literature^3,41,42^, among which we adopted the one proposed by Müller‐Trede et al.^3^; we chose this method because the decomposition consisted of psychologically meaningful factors. The MSE decomposition of Müller‐Trede et al.^3^ is represented as follows:

1 $$MSE \, = \, Bias \, + \, Variability \, bias \, + \, Linear \, correspondence$$

First, *bias* represents the degree of difference in the rating value of a Giver, compared to that of a Receiver. Simply put, it was larger when the mean rating value of a Giver differed more from that of a Receiver. Second, *variability bias* indicates the degree of difference in the variability of the Giver’s opinions from the optimal degree of regression to the mean. Specifically, variability refers to the standard deviation of the Giver’s rating values across stimuli. Concerning the regression to the mean, we multiplied the variability of the Receiver’s opinions using a Giver–Receiver’s correlation coefficient across the stimuli (see mathematical description in ‘Methods’). Third, *linear correspondence* denotes the extent to which a Giver–Receiver correlation deviates from a linear relation.

Table 2 shows the results of the MSE decomposition. Remarkably, Blended opinion recorded lower values in variability bias than Own opinion, across two studies (95% CI). We did not find such results concerning bias and linear correspondence (see also Supplementary Fig. S1). The results indicated that our method was effective because of the improvement of the regression to the mean. Notably, the results were in line with the findings on the wisdom of the crowd for matters of taste (Müller-Trede et al.^3^). Considering this perspective, we can regard our method as exploiting the wisdom-of-crowd effect for matters of taste on a within-person level. In the Discussion section, we address this study’s contribution to wisdom-of-crowd literature.

**Table 2:** Results of the MSE decomposition (95% CI; in Study 1, all types of opinions did not follow a normal distribution, *Kolmogorov–Smirnov* test: *ps* < 0.001). It can be observed that the Blended opinion had a smaller *variability bias* than the Own opinion across the two studies. In addition, in Study 1, the Blended opinion had a smaller *bias* and *variability bias *than the Estimated opinion.

Study 1	Bias	Variability bias	Linear correspondence
Own	[641.79, 727.88]	[351.34, 415.41]	[283.72, 300.39]
Estimated	[787.48, 884.64]	[350.01, 407.73]	[275.79, 291.46]
Blended	[609.26, 690.30]	[274.15, 315.90]	[270.67, 286.38]

Study 2	Bias	Variability bias	Linear correspondence
Own	[118.91, 179.41]	[207.32, 303.39]	[449.15, 479.40]
Estimated	[98.52, 124.97]	[86.37, 162.80]	[447.09, 472.86]
Blended	[95.76, 126.20]	[88.53, 145.54]	[432.29, 454.59]

In Study 1, Blended opinion had both lower bias and variability bias, compared with Estimated opinion. The results indicated that the rating values of Estimated opinion were consistently farther from those of Own opinion, compared with Blended opinion (higher, most often; see also Table 1). This result was not found in Study 2.

### Additional analysis: when is our method more (or less) effective

As an additional analysis, we investigated the conditions under which our method performed better (or worse). We focused particularly on two factors: individual differences and taste discrimination.

#### Individual differences

There are diverse types of tastes among people^3,19,43,44^: Some have considerably different tastes from the general public, while others have ordinary ones. Here, we examined how individual differences in terms of taste typicality influenced the efficacy of our method.

**Figure 5:**
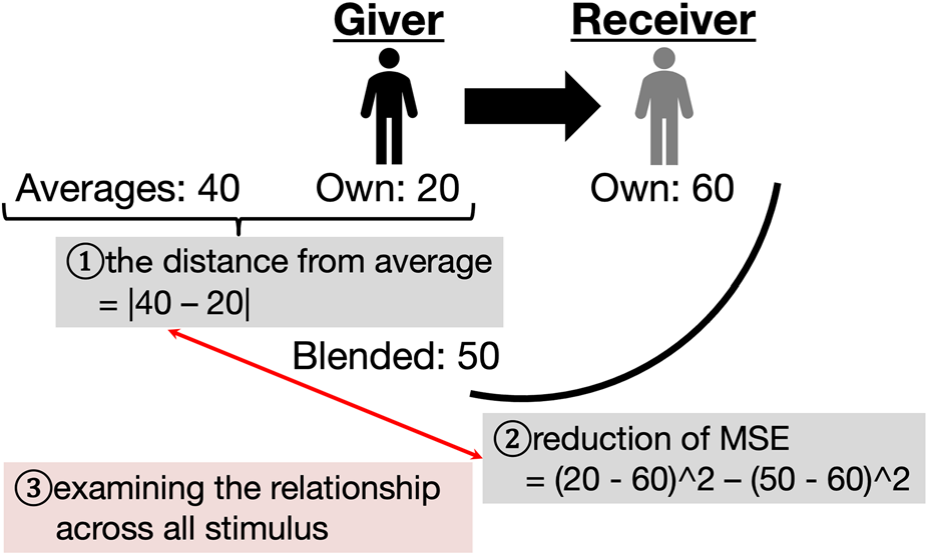
Illustration of the analysis in the ‘Individual differences’ Section. (1) We first calculated the ‘distance from the average’, which indicates the taste typicality of a Giver. Specifically, we computed the absolute distance between the Giver’s Own opinion and the averages of all Own opinions. (2) Subsequently, we analysed the ‘reduction of the MSE (Mean Squared Error)’ to use our method for all stimuli. This was calculated by subtracting the case when the Giver’s opinion was a Blended opinion from when it was an Own opinion. (3) Finally, we examined the relationship between the distance from the average and the reduction in MSE. In particular, we calculated the correlation coefficient between them. We assigned all participants, except the Giver, to Receivers and performed this analysis. Additionally, we assigned all participants to Givers and conducted the same procedure.

Figure 5 illustrates our analysis. (1) For the typicality of the tastes of a Giver, we calculated the absolute distance between the Giver’s and all participants’ Own opinions (this means the averages of all participants, called ‘distance from average’); that is, a small distance from average value represents high typicality of the Giver’s taste. (2) We then analysed the reduction of the MSE. This was calculated by subtracting the value of when the Giver’s opinion was Blended from when it was Own; the larger the reduction of MSE, the better the performance of our method. (3) We conducted this analysis across all stimuli and examined the relationship between the distance from the average and the reduction of MSE. Specifically, we calculated the correlation coefficient between them.

The following procedure was the same as in the final paragraph of the ‘Analysis’ section: for the Giver, we performed this analysis across the Receivers (all participants except the Giver). The averages of the reduction in the MSE were then assigned to the Giver’s value. Finally, we conducted this procedure for all the participants.

**Figure 6:**
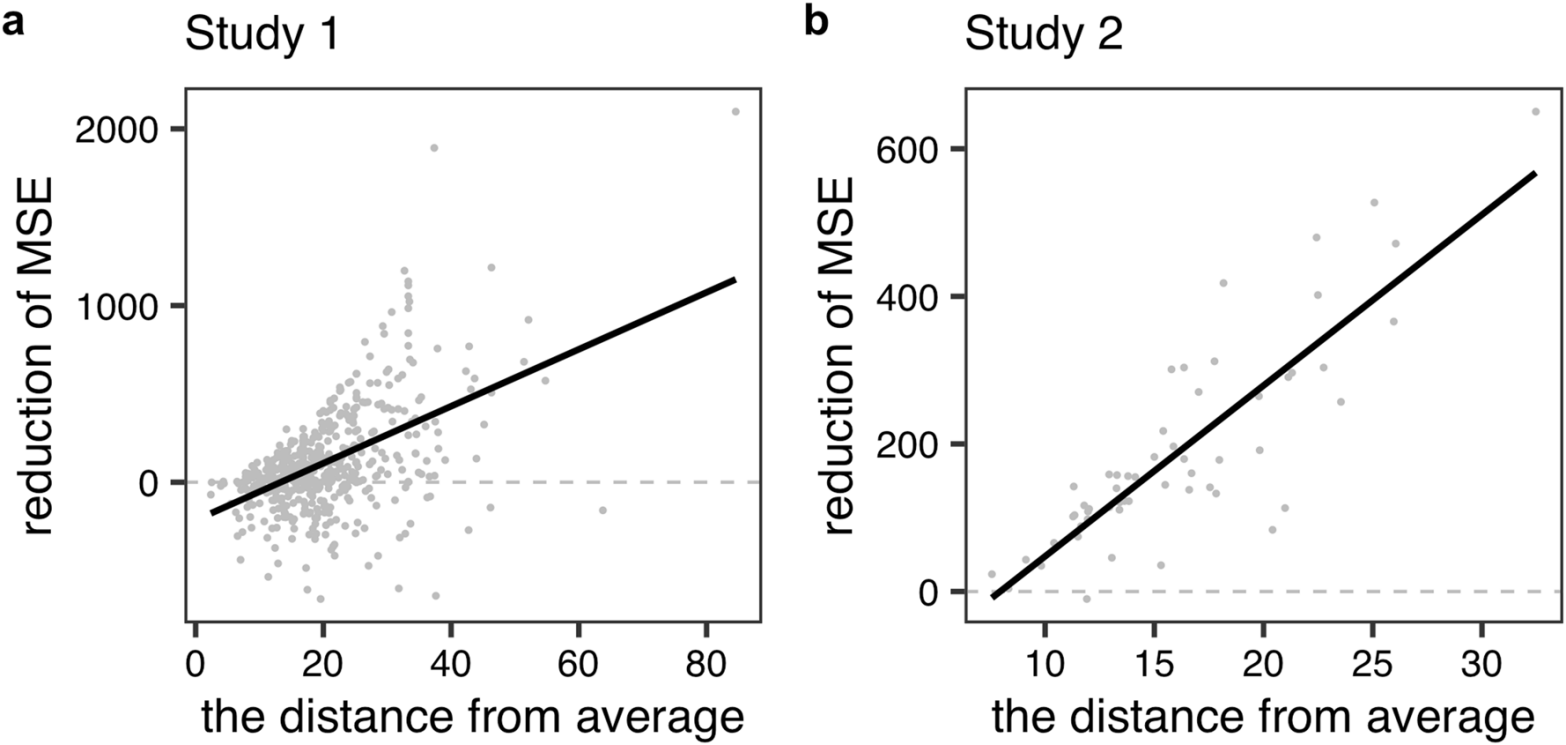
Results of the ‘Individual differences’ section in Study 1(**a**) and Study 2(**b**). The black lines represent the regression lines. The larger the distance from the average, the larger the reduction of the MSE.

Figure 6 shows the results of the analysis. Each plot indicates the Giver’s value. The *x*-axis represents the distance from average, while the *y*-axis represents the reduction of the MSE. Across the two studies, we found a significant positive relationship between them (Study 1: *rho* = 0.42, *p* < 0.001; Study 2: *r* = 0.67, *p* < 0.001). That is, for a Giver with atypical taste, our method was more effective (for further analysis, see Section S2 of the Supplementary Information).

#### Taste discrimination

When assessing items in our daily lives, we have different feelings concerning our evaluations. For some items, we are able to make distinctive judgements about whether we like them or not (for example, pop and metal music), while for other items, we can only make vague judgements (for example, ambient and experimental music). Previous studies^1,3^ indicate that the distinctiveness of judgements (called ‘taste discrimination’) plays a critical role in opinion giving. Notably, Müller-Trede et al.^3^ show that taste discrimination affects the helpfulness of opinions in our context. They primarily provide a theoretical model and point out the effects of taste discrimination; briefly, they define taste discrimination based on signal-to-noise ratios on judgements. Subsequently, they performed behavioural research and empirically confirmed its influences. Specifically, familiarity with the stimulus to which participants responded was used as an index of taste discrimination.

We therefore investigated the effect of taste discrimination on the effectiveness of our method. In this section, we only utilised the behavioural data from Study 2, in which the participants indicated Own opinion and Familiarity, identical to the study of Müller-Trede et al.^3^. We also provided ‘Difficulty’ as a new index for taste discrimination: Participants directly answered how challenging they found it to answer the Own opinion (see more details in ‘Methods’).

We conducted mixed-effects analyses that included the ‘Reduction of MSE’ as a dependent variable and Familiarity, Difficulty, and the interaction term as independent variables (Table 3). The impact of Difficulty was significant (*F*(1, 1123.7) = 48.32, *p* < 0.001). Further, we found no effect of Familiarity or interaction (Familiarity: *F*(1, 427.1) = 0.13, *p* = 0.72; interaction term: *F*(1, 1311.3) = 0.90,* p* = 0.34). This shows that, concerning our experimental settings, only Difficulty influenced the effectiveness of our method. Subsequently, findings showed that the lower the Difficulty, the higher the efficacy of our method: *reduction of the MSE* =  − 3.23 × *Difficulty* + *intercept* (= 288.21).

**Table 3:** Results of the GLMM (a Generalized Linear Mixed Model). * indicates the interaction term.

Independent variable	Statistics
Difficulty	*F* (1, 1123.7) = 48.32, *p* < 0.001
Familiarity	*F* (1, 427.1) = 0.13, *p* = 0.72
Difficulty * Familiarity	*F* (1, 1311.3) = 0.90, *p* = 0.34

Next, we determine how lower difficulty enhanced our method. To examine this issue, we performed separate additional mixed-effects analyses for Own and Blended opinions (Tables 4 and 5, respectively). These analyses used the same independent variables as the previous analysis: Familiarity, Difficulty, and interaction term. However, it included a different dependent variable: the MSE (not the reduction of the MSE).

**Table 4:** Results of the additional GLMM for Own opinion.

Independent variable	Statistics
Difficulty	*F* (1, 1298.4) = 92.16, *p* <0 .001
Familiarity	*F* (1, 1211.0) = 1.28, *p* = 0.26
Difficulty * Familiarity	*F* (1, 1329.5) = 1.96, *p* = 0.16

**Table 5:** Results of the additional GLMM for the Blended opinion.

Independent variable	Statistics
Difficulty	*F* (1, 1286.1) = 58.01, *p* < 0.001
Familiarity	*F* (1, 1245.0) = 0.16, *p* = 0.69
Difficulty * familiarity	*F* (1, 1326.8) = 3.11, *p* = 0.078

The results showed that the effects of Difficulty were significant (*ps* < 0.001), and neither Familiarity nor interactions were significant (all *ps* > 0.1), for both Own and Blended opinions. Concerning Difficulty, the MSEs in both Own and Blended opinions had inverse relationships: as Difficulty increased, the reduction in MSE decreased. Importantly, Own opinion had larger slopes than Blended: *Own* =  − 6.48 × *Difficulty* + *intercept* (= 1034.49), and *Blended* =  − 3.03 × *Difficulty* + *intercept* (= 739.58). That is, as Difficulty increased, Own opinion rapidly became an accurate prediction. Consequently, the merits of using our method were relatively low when people found it challenging to answer their own opinions.

## Discussion

This study proposed a method for improving an individual’s prediction of others’ future satisfaction. This method requires the individual to evaluate an item twice, from different viewpoints; one would state their own preferences, while the other would estimate public opinion^26,35–40^. Using two behavioural studies and computer simulations, we comprehensively examined our proposed method. We first confirmed its effectiveness; by averaging the two opinions, an individual could improve their predictions (concerning optimal weightings on the opinions, see Section S4 of the Supplementary Information). Subsequently, we mathematically analysed^3^ our method to determine how it performed effectively. Moreover, we identified multiple factors that influenced the efficiency of our method.

As mentioned in the Introduction, previous studies^2,3,19^ demonstrate that the wisdom-of-crowd effect could emerge in terms of matters of taste *within a group*. In contrast, this is the first study that shows that the wisdom-of-crowd effect for matters of taste could emerge even *within a person* (a related study^4^ focuses on performance evaluation as a kind of wisdom of the crowd for matters of taste, and extends it to a within-person level). Specifically, the analysis on decomposing MSE indicated that the same mechanism worked for the wisdom-of-crowd effect for matters of taste – both within a group and within a person. It functions mainly by reducing variability bias (in other words, improving the regression to the mean).

The remaining question is how effective our method was. To answer this, we conducted an additional analysis that compared our method (i.e. one individual’s Blended opinion) with two individuals’ Own opinions (see Supplementary material, Section S5). As a result, our method recorded relatively high efficacy for the wisdom-of-inner-crowd method, especially in Study 2 (1.92 individuals; 1.36 individuals in Study 1). For matters of *fact*, most previous studies^20,23,27^ record approximately 1.1–1.3 individuals. Therefore, this high efficacy may be a characteristic for matters of *taste*. However, it should be added that the results may be due to the settings of our method (i.e. estimating public opinion).

It must be noted that academically, we can associate our study with differential-information theories^45,46^ and social sampling^36,37,47,48^. These studies suggest that when people estimated public opinion, their opinions were based on themselves or on a similar social circle. Therefore, in our context, would people with atypical taste have a poorer sense of what the average is? The answer is yes; to address this question, we conducted an additional analysis. We examined the relationship between the distance from average and prediction accuracy for the average (i.e. how different is a Giver’s Estimated opinion from the average of all people’s Own opinions?). We found that people with more atypical tastes have a poorer sense of what the average is, across the two studies (Study 1: *r* = 0.47, *p* < 0.001; Study 2: *r* = 0.58, *p* < 0.001; see also Supplementary Fig. S5).

This study also fits in with the advice-taking paradigm^49^. However, it should be noted that this study conducted advice-taking *automatically.* That is, for our method, we systematically averaged a giver’s Own and Estimated opinions, and directed it to a Receiver. In this respect, the existing findings^50^ on advice-taking show that a Receiver usually does not average two opinions naturally (e.g. a Receiver adopts only one of the two opinions). It would therefore be a challenge to investigate whether the Receiver naturally averaged the Giver’s two opinions, as in our method.

In terms of practical contributions, this study may contribute to the online interface. It is well-known that items on online review sites often get very few reviews. For example, half of the items on Amazon.com only get one review^51^. In these circumstances, the helpfulness of the reviews could improve if the reviewer used our method. Reviewers would, of course, need incentives to use our method. However, this paper suggests how to gather more helpful opinions beyond people’s own opinions, at the least.

Our method could also contribute to research on the recommender system. Previous studies^19,52^ on the recommender system show that an individual could leverage the experiences of similar others. Conversely, as the Estimated opinion differed from the Own opinion, an individual might learn from people with dissimilar tastes, when applying our method.

One limitation of this study is related to the efficacy of the method. As mentioned above, our method recorded relatively high efficacy for the wisdom-of-inner-crowd method. However, our method (i.e. Blended opinion) could not defeat the combined opinion of two individuals (i.e. two Own opinions) across two studies. Subsequently, more sophisticated methods should be investigated in future. One example is increasing the number of Estimated opinions (e.g. four times) and combining it with other wisdom-of-inner-crowd methods (e.g. introducing timespan^20^).

In terms of other future studies, one promising approach is related to ‘Difficulty’. Based on an existing theoretical framework^3^, we asked participants to report difficulty level and found that the lower the difficulty was, the less effective our method was. How did these results emerge? One possible explanation is that when an individual felt it difficult to rate an item, the individual might find it highly difficult to project the average for other users. This means that the individual was off the mark in Estimated opinion (e.g. 100 in Study 1), resulting in the obtained results. In the future, we aim to test this explanation. For simplicity, we plan to ask participants about the difficulty of producing an Estimated opinion directly.

Another promising future research direction is determining the efficacy of our method; specifically, we aim to compare our method with two ‘own opinions’ or two ‘estimated opinions’. We plan to examine two ‘own opinions’ using open access data from a previous study^53^ as the first step.

Overall, we consider that this study highlights the generality of the wisdom-of-crowd phenomenon. That is, we can exploit the wisdom-of-crowd effect without objective criteria and multiple people.

## Methods

**(1) Details of the experiment in Study 1.**

We recruited 543 Japanese adults (273 females and 270 males, *M*_*age*_ = 45.23 years, *SD*_*age*_ = 11.01 years) to participate in the experiment through a web research company. All participants provided informed consent prior to study enrolment. The experimental protocol was approved by the University of Tokyo Research Ethics Committee and conducted in accordance with the latest version of the Declaration of Helsinki. Participants received cash-equivalent points that can be used for online shopping in Japan as an incentive.

We set five conditions for this research. In each condition, five different paintings were utilised as stimuli. Their contents varied among the five conditions. As a result, the stimulus consisted of 25 paintings in total (= *5 conditions* × *5 paintings*; Supplementary Table S3). Regarding the selection of the paintings, we followed a method used by a previous study^54^ and included various paintings such as Gothic, Renaissance, Surrealism, and Modern art. In the experiments, the participants were randomly assigned to one of the five conditions and asked to evaluate the paintings. Specifically, they were asked the following two questions: ‘How much would you like to hang this picture on your wall?’^55^ (Own opinion) and ‘How much would the average people like to hang this picture on their wall?’ (Estimated opinion). We randomised the order of the paintings’ presentation for each participant. Moreover, for convenience, we first analysed each condition, after which we combined the results across the conditions.

**(2) Details of the experiment in Study 2.**

We recruited 56 Japanese undergraduate and graduate students (22 females and 34 males, *M*_*age*_ = 19.61, *SD*_*age*_ = 1.40) for the second experiment. All participants provided informed consent prior to study enrolment. The experimental protocol was approved by the University of Tokyo Research Ethics Committee and conducted in accordance with the latest version of the Declaration of Helsinki. On its completion, they received a flat fee of 1,000 Japanese Yen (approximately US$ 9.17 at the currency rate at the time of the experiment).

The experimental settings were the same as those employed by Müller-Trede et al.^3^. In this study, we set only a single condition: All participants followed the same experimental procedure and evaluated identical stimuli. They were asked the following two questions: ‘How much do you like the musical piece?’ (Own opinion) and ‘How much would the average people like the musical piece?’ (Estimated opinion).

We selected songs from various music genres, such as classic, folk, hip-hop, and ethnic. Specifically, we selected two songs each from twelve musicians (for example, two songs by Oasis from the same album ‘What’s the Story Morning Glory?’; see Supplementary Table S4). Thus, the stimuli consisted of 24 songs. One minute of each song was presented to participants. We randomised the order of the musical pieces for each participant.

Furthermore, this research involved two additional questions: Difficulty in answering the Own opinion (‘How difficult was it for you to state your preference?’) and Familiarity with the song’s genre (‘How familiar are you with the genre of this musical piece?’). Participants responded to these questions on a scale ranging from 0–100.

**(3) A formula of MSE decomposition.**

As mentioned in the Results section, the MSE decomposition proposed by Müller‐Trede et al.^3^ is represented as Eq. (1).

Mathematically, in our context, the MSE decomposition was represented as follows:

2 $$MSE_{Giver, \, Receiver} = \, (M_{Giver} - M_{Receiver} )^{2} + \, (\sigma_{Giver} - \rho_{Giver, \, Receiver} \times \sigma_{Receiver} )^{2} + \, (1 - \rho_{Giver, \, Receiver}^{2} )\sigma_{Receiver}^{2}$$

*M* is the mean of the rating value, *σ* is its standard deviation, and *ρ* is the correlation between a Giver’s and a Receiver’s ratings. On the equation’s right-hand side, the first, second, and third terms correspond to *bias*, *variability bias*, and *linear correspondence*, respectively.

**(4) Assumptions in our analysis.**

The analysis supposed that we could measure the difference in the tastes between the Givers and Receivers on a discrete scale. Many studies^3,4,8,56–58^ that explored preference predictions have also made similar assumptions.

**(5) Mixed-effects analysis.**

We performed all mixed-effects analyses using the *R* packages *lme4* and *lmerTest*^59^. In particular, we selected the best model and computed all statistical values using *the step()* function for the full model, with random participants and stimulus intercepts.

## Supplementary Information

Supplementary Information.

## Data Availability

The R-code during the current study and the two datasets analysed during the current study (including data for creating the figures) are available in the Mendeley Data: 10.17632/tr952fsrpx.1.
