# Supplementary material for: A simple cognitive method to improve the prediction of matters of taste by exploiting the within-person wisdom-of-crowd effect
Source: Sci Rep. 2022 Jul 20;12:12413. doi: 10.1038/s41598-022-16584-7 (PMC9300593; doi:10.1038/s41598-022-16584-7)
Supplement: Supplementary file 1 — Supplementary Information. [file 41598_2022_16584_MOESM1_ESM.docx]

Supplementary information

A simple cognitive method to improve the prediction of matters of taste by exploiting the within-person wisdom-of-crowd effect

*Itsuki Fujisaki^1^, Hidehito Honda^2^, & *Kazuhiro Ueda^1^

^1^ Graduate School of Arts and Sciences, The University of Tokyo; Tokyo, Japan

^2^ Faculty of Psychology, Otemon Gakuin University; Osaka, Japan

*e-mail: bpmx3ngj@gmail.com (I.F.), ueda@gregorio.c.u-tokyo.ac.jp (U.K.)

**This PDF file includes:**

Supplementary text

Figures S1 to S10

Table S1 to S5

**Fig S1. Results of the MSE decomposition in the two studies**

**
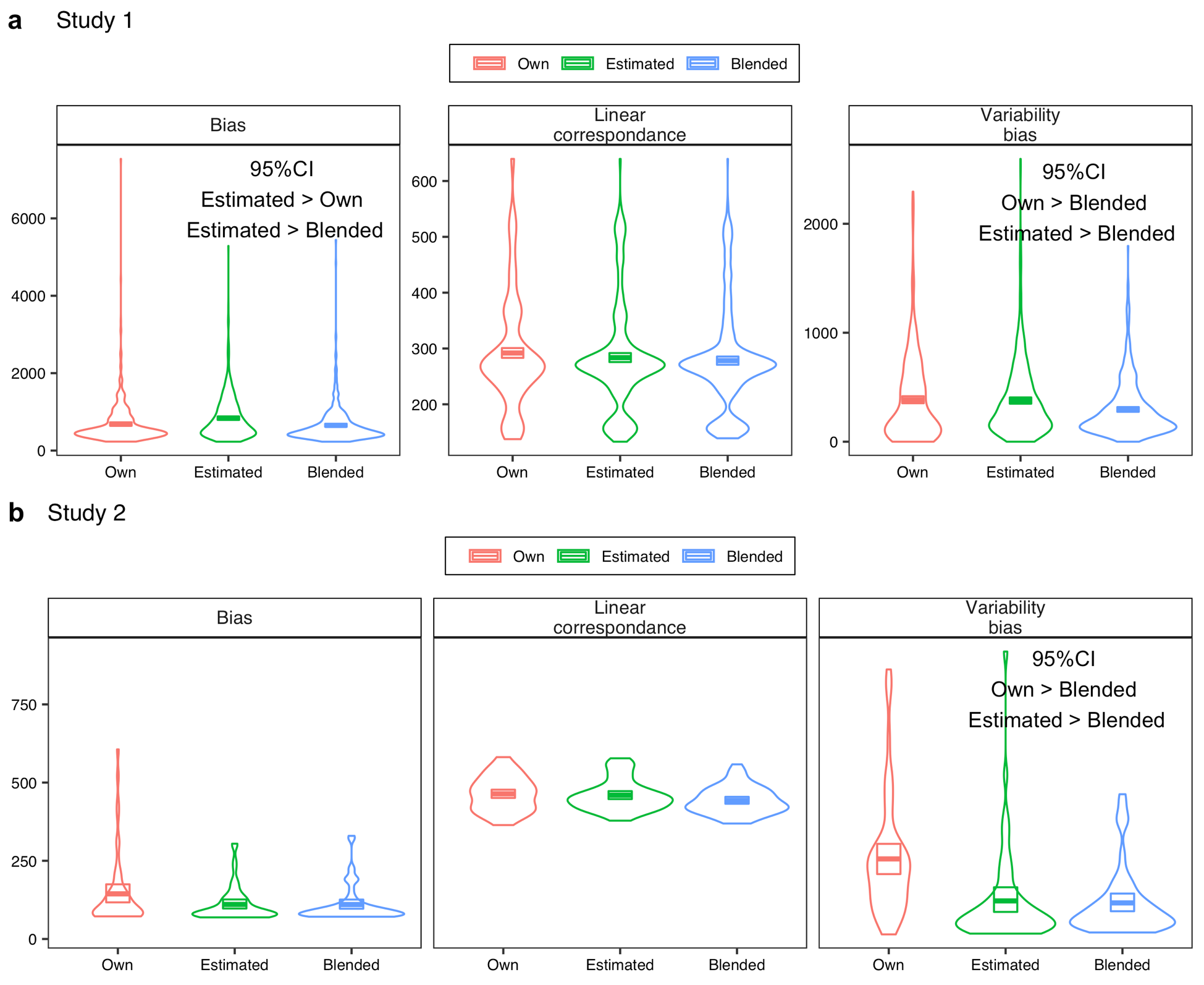
**

Fig. S1. Results of the MSE decomposition in Study 1(a) and Study 2(b).

**S2. Additional analysis on the individual differences**

In ‘Further analysis: Individual differences’, we focused on the ‘taste typicality’ of Givers and found that it influenced the effectiveness of our method.

Here, for an additional analysis, we examined the effect of Givers’ ‘taste variability’: some Givers had a low variability in their Own opinions (for example, they answered about 60 for all stimuli), whereas others had a high variability (for example, they responded with a score of 100 or 0). Does taste variability affect the effectiveness of our method?

To address taste variability, we calculated the variance of the ratings for all stimuli. We then conducted a multiple regression analysis that included the reduction of the MSE as a dependent variable, as well as taste typicality (using ‘Distance from the Average’, as in the main text), taste variability, and the interaction term as independent variables.

The results demonstrated that only the distance from the average was significant (*p* < .01), while the others were non-significant (taste variability: *p* = .43; interaction term: *p* = .58). As discussed in the main text, taste typicality is therefore the key to our method’s efficiency.

**S3. Further analysis: taste similarity**

This section focuses on the Giver–Receiver pair. Specifically, we examined the influence of *taste similarity*^1–3^; some pairs have similar tastes, while others have different ones. Previous studies^1–3^ demonstrate that the taste similarity between a Giver and a Receiver plays a significant role in opinion-giving. We therefore investigated how taste similarity affected the effectiveness of our method.

We only utilised the data from Study 2 to perform the following analysis. The stimuli consisted of 24 songs (*12 musicians* × *2 songs*). Each song was trimmed to a one-minute musical piece. We divided the experimental stimuli into two sets for further analysis, each of which included 12 musical pieces from 12 musicians. Across the two sets, the order of the musicians remained constant.

In Set 1, we examined taste similarity as follows: First, all participants except the Giver were treated as Receivers. We then calculated taste similarity for each Giver–Receiver pair. We accomplished this by calculating a correlation coefficient between the Givers’ and the Receivers’ Own opinions across the stimuli in Set 1. We then ranked the Receivers according to the correlation coefficients (in descending order). In Set 2, we investigated the effectiveness of our method by computing the reduction of the MSE for each pair, as mentioned in the ‘Individual differences’ section. This procedure was performed for all participants.

Fig. S2 displays the results of our analysis. The *x*-axis represents the rank of the taste similarity, whereas the *y*-axis represents the reduction in MSE. Each plot indicates the rank of the taste similarity. Each value in the reduction of the MSE is the average across all Givers. We found a significant positive association between them (*rho* = 0.90, *p* < .001): the higher the rank of the taste similarity, the larger the reduction in MSE. Therefore, our method worked more effectively for Giver–Receiver pairs with different tastes.

It should be noted that the results also indicated that, in all cases, our method worked to some extent, irrespective of taste similarity. As shown in Fig. S2, in all ranks of taste similarity, the reduction in MSE had positive values (particularly above 100).


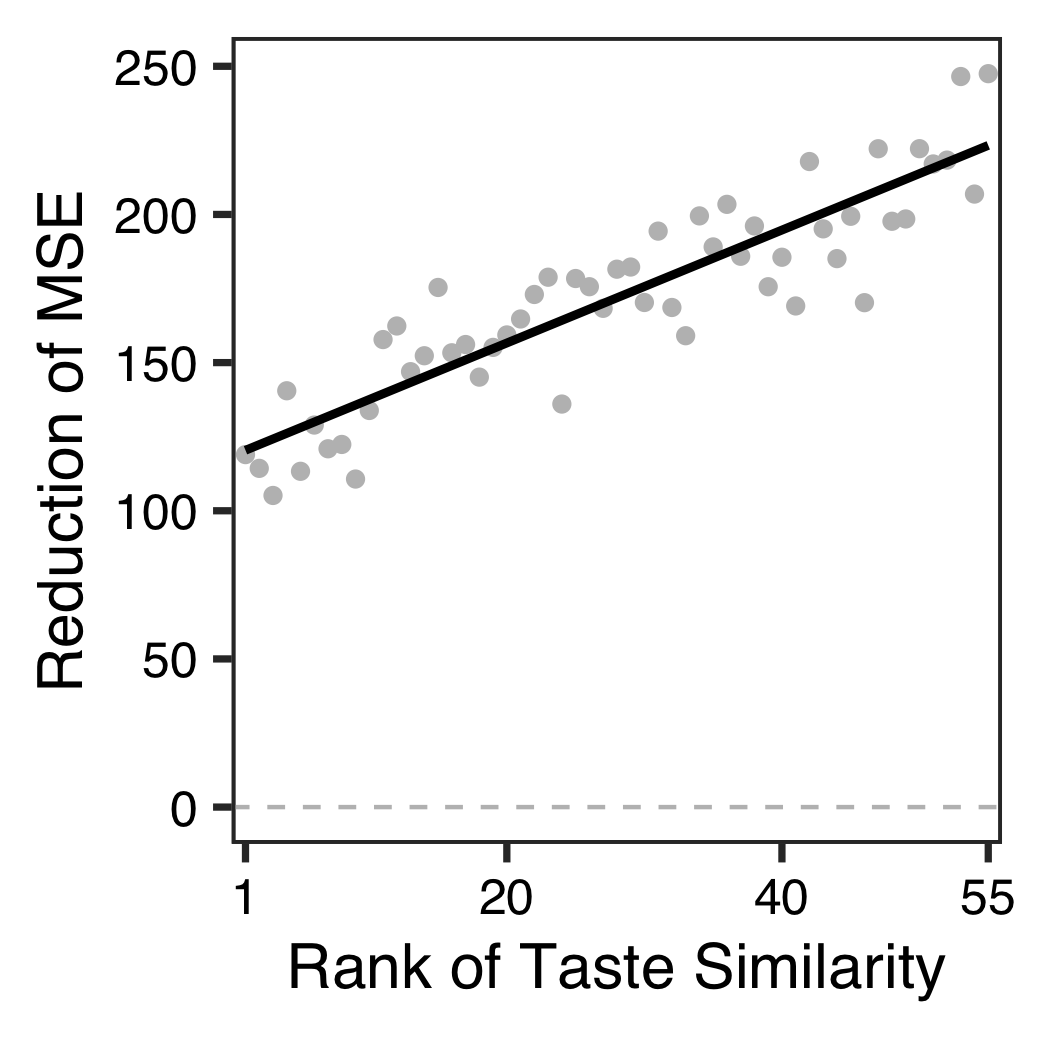


Fig. S2. Results of the additional analysis: taste similarity. The figure shows the relationship between the rank of taste similarity and the reduction of MSE. The black line represents the regression line. We observe that the higher the rank of taste similarity (that is, a Giver-Receiver’s pair showed different tastes), the larger the reduction of MSE. The grey dotted line denotes when the reduction in MSE is zero (that is, there are no merits to using our method). As shown in the figure, all ranks of taste similarity are above zero.

**S4. Optimal weighting**

Here, we calculated the optimal weighting of Own and Estimated opinions using the following equation:

(*w* × Giver_Own_ + (100 - *w*) × Giver_Estimated_) / 100

where *w* represents the weighted percentage of a Giver’s Own opinion. We manipulated *w* from 0 to 100, one step at a time. Specifically, we set 101 levels (*w* = 0, 1.... .99, and 100) and calculated the MSE for each *w*.

Fig. S3 shows the results, which indicate the relationship between *w* and MSE. The red dotted line represents optimal weighting: in Study 1, *w* = 60 and in Study 2, *w* = 30. Specifically, which opinion should be weighed more depended on the study (Study 1: Own; Study 2: Estimated).

*
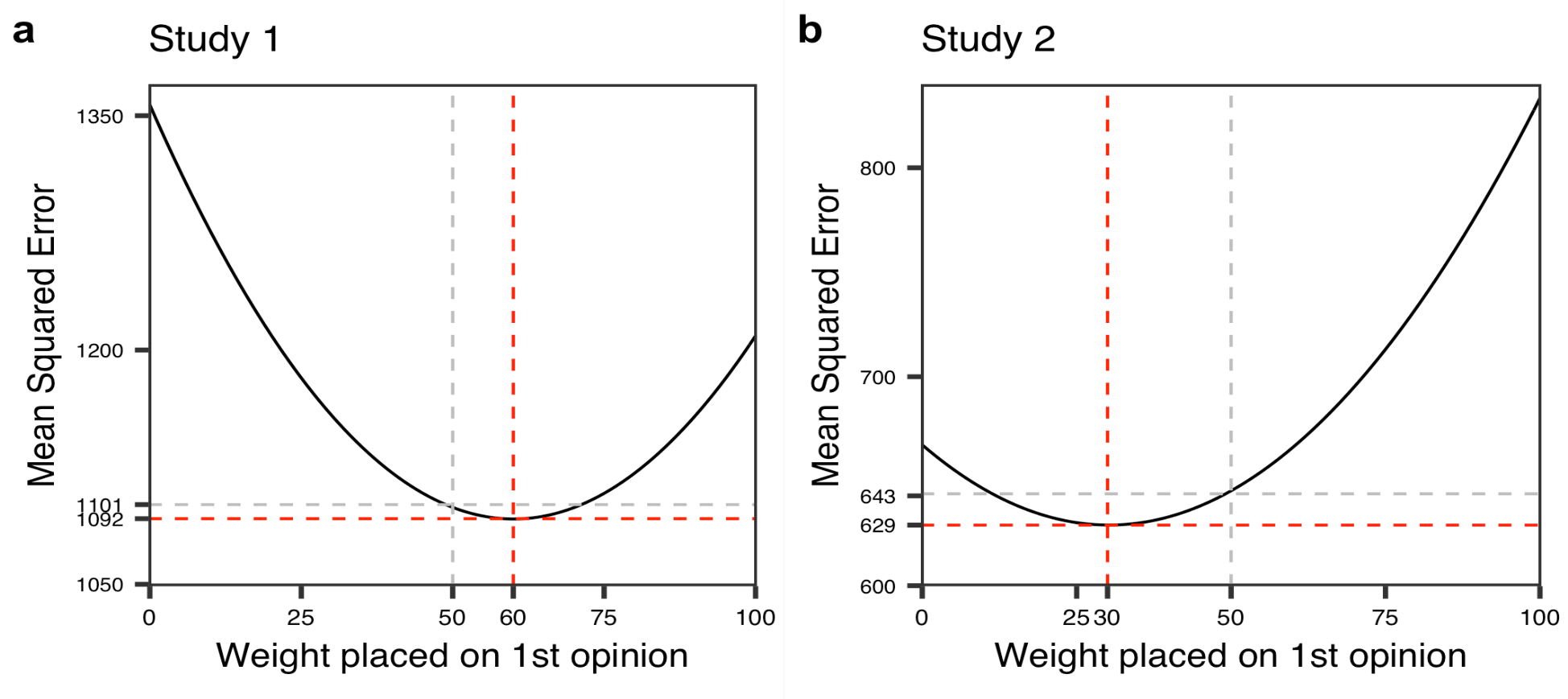
* The figure also shows the dotted grey line for Blended opinion (*w* = 50). We observe that these MSEs are almost the same as the optimal weighting across the two studies (Study 1: 0.82%; Study 2: 2.38% higher). Overall, our method seems to be a robust strategy.

Fig. S3. Results of the optimal weighting in Study 1(a) and Study 2(b)

**S5. Results of the additional analysis for two people in the two studies.**


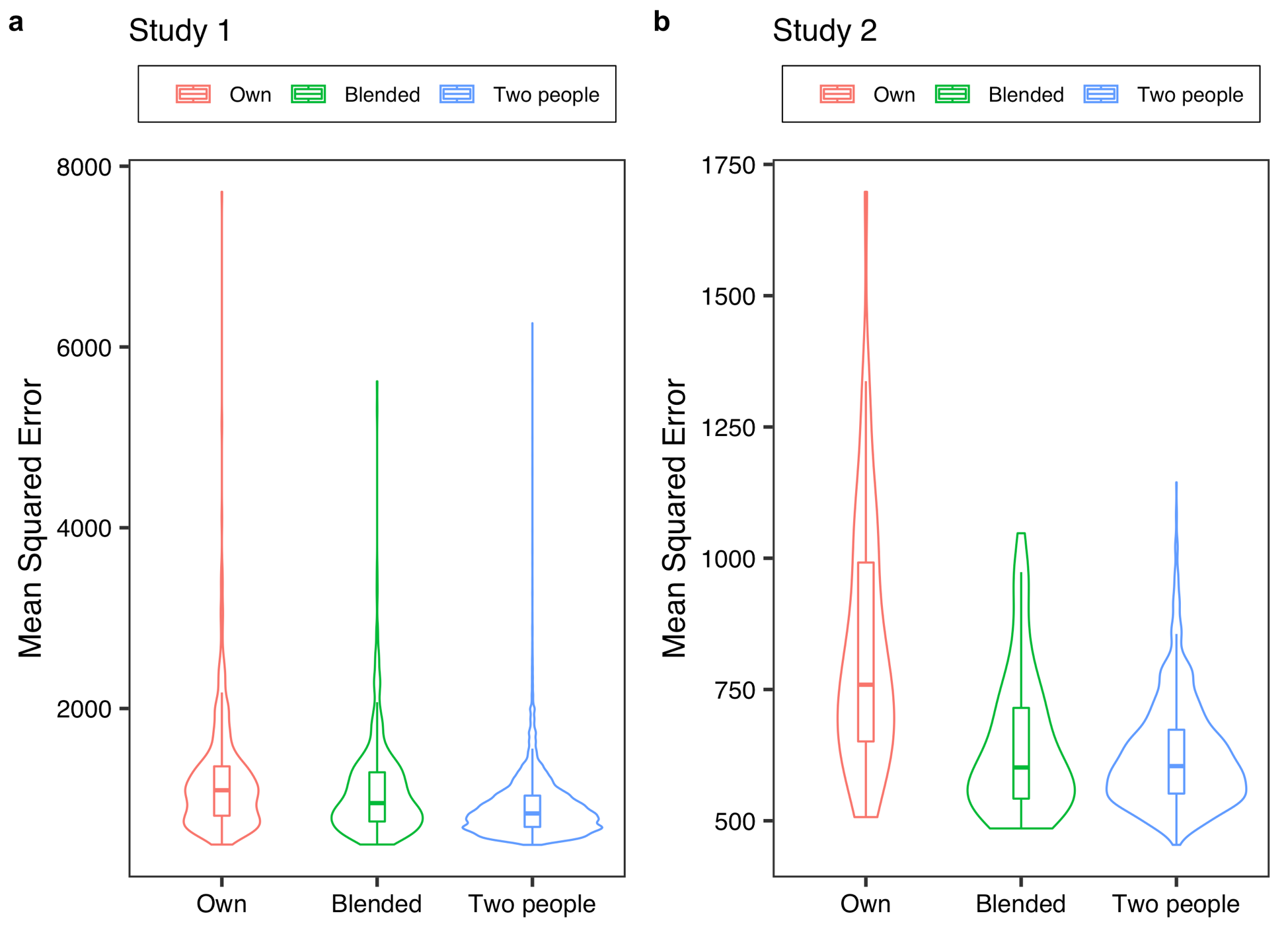


Fig. S4. Results of the analysis in Study 1(a) and Study 2(b). In two people, we first computed the averaged value of their Own opinions and then calculated the MSE

Table S1. Average values of the MSE

|  | Own | Blended | Two people |
| --- | --- | --- | --- |
| Study 1 | 1208.64 | 1098.96 | 906.13 |
| Study 2 | 883.27 | 645.42 | 624.95 |

**
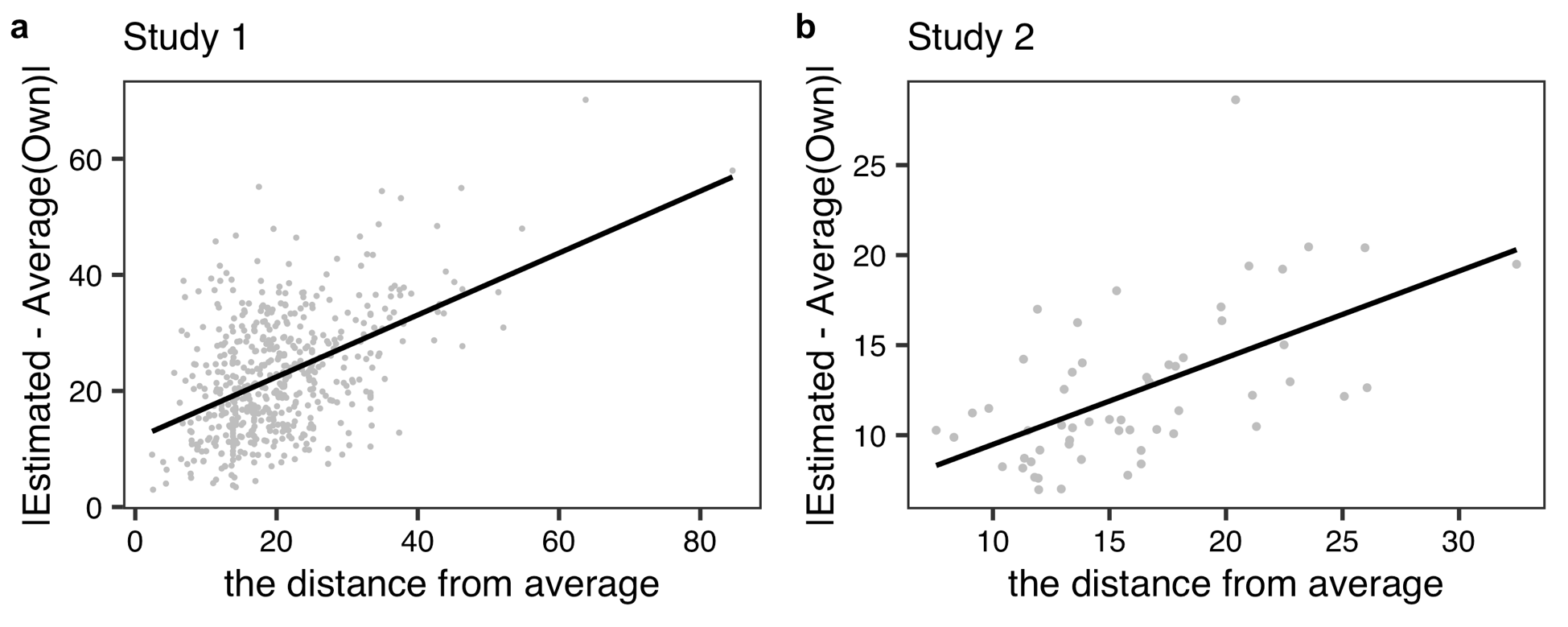
S6. The relationship between the distance from average and prediction accuracy for the average**

Fig. S5. The results of the analysis. The black lines indicate the regression lines.

**S7. Analysis of receivers’ side**

This section analyses the effect of inter-individual differences on receivers. We focused on whether our method worked equally for receivers with typical and atypical tastes. In other words, we investigated the relationship between the efficacy of our method and a receiver’s taste typicality.

Fig. S6 shows the results. We calculated the reduction of MSE and the distance from average as in the ‘Further analysis: when does our method works better (or worse)’ section in the main text. In Study 2, a reduction of MSE recorded a positive value, irrespective of the distance from average (*p* = 0.38; *r* = -0.11). On the contrary, in Study 1, a reduction of MSE correlated with the distance from average (*p* < .01; *r* = 0.51) and recorded a negative value when the value on the distance from average was small (e.g., 5). That is, the receiver who has typical taste might as well not receive an opinion using our method.

How did the results emerge? As Fig. 4(a) shows, in Study 1, the rating values of Own opinion focused on 0. Thus, when the distance from the average was small, the rating value of Own opinion was around 0. Subsequently, the MSE of Blended opinion became larger than the MSE of Own opinion, as Blended opinion had a systematically larger rating value than Own.

Conversely, in Study 2, this tendency (i.e., the rating values of Own opinion focused on 0) was not observed. As Fig. 4(b) indicates, Own and Blended opinion both followed a normal distribution and Blended opinion had smaller variance than Own opinion. Subsequently, irrespective of the rating value of a Receiver’s Own opinion, a Giver’s Blended opinion did not tend to have larger value than a Giver’s Own opinion (for example, the value of Receiver was 5 and that of Giver was 95).


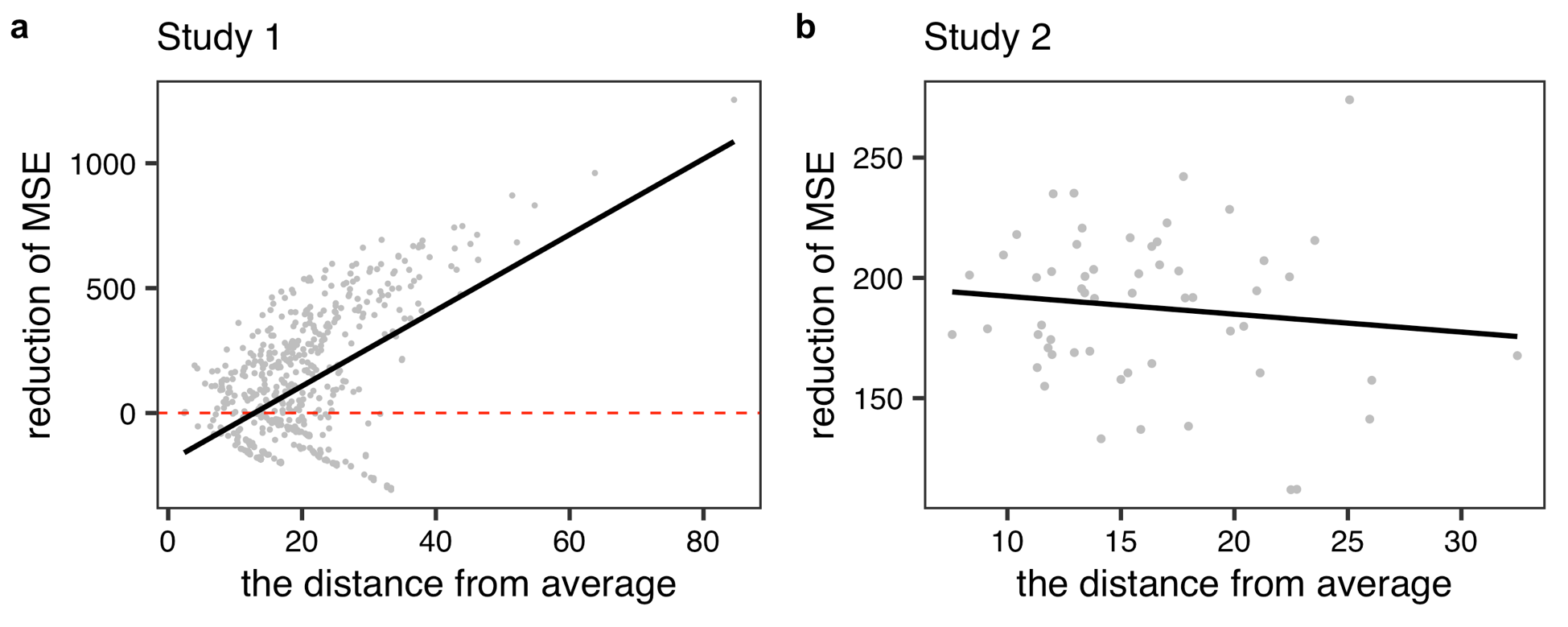


Fig. S6. The results of the analysis in Study 1(a) and (b). The black lines represent the regression lines. The red dotted line indicates that our method did not work (i.e., reduction of MSE was 0).

**S8. Further analysis: when did our method work better?**

In this section, we examined the conditions in which our method worked better, especially focusing on the rating values.

We employed a generalized linear mixed model (GLMM) that included the improvement by using our method (that is, MSE_Own_ − MSE_Blended_) as a dependent variable, the rating values of Own, Estimated, and the interaction term as independent variables, and participants and questions as random factors.

The results showed certain tendencies (Table S1-2): In Study 1, our method would improve when the rating value of Own was large, that of Estimated was small, and the interaction was small (*ps* < .01). In Study 2, our method would improve when the rating value of Estimated was large, and the interaction was small (*ps* < .01).

| Independent variable | Estimate |
| --- | --- |
| Own | 8.66 (*p* < .001) |
| Estimated | -4.59 (*p* < .001) |
| Own * Estimated | -0.12 (*p* < .001) |

Note that this is a brief analysis. In the future, we aim to gather large datasets for a detailed analysis.

Table S3. Results of the GLMM in Study 2.

Table S2. Results of the GLMM in Study 1. * indicates the interaction term.

| Independent variable | Estimate |
| --- | --- |
| Own | 1.36 (*p* = 0.26) |
| Estimated | 4.29 (*p* < .001) |
| Own * Estimated | -0.073 (*p* < .001) |


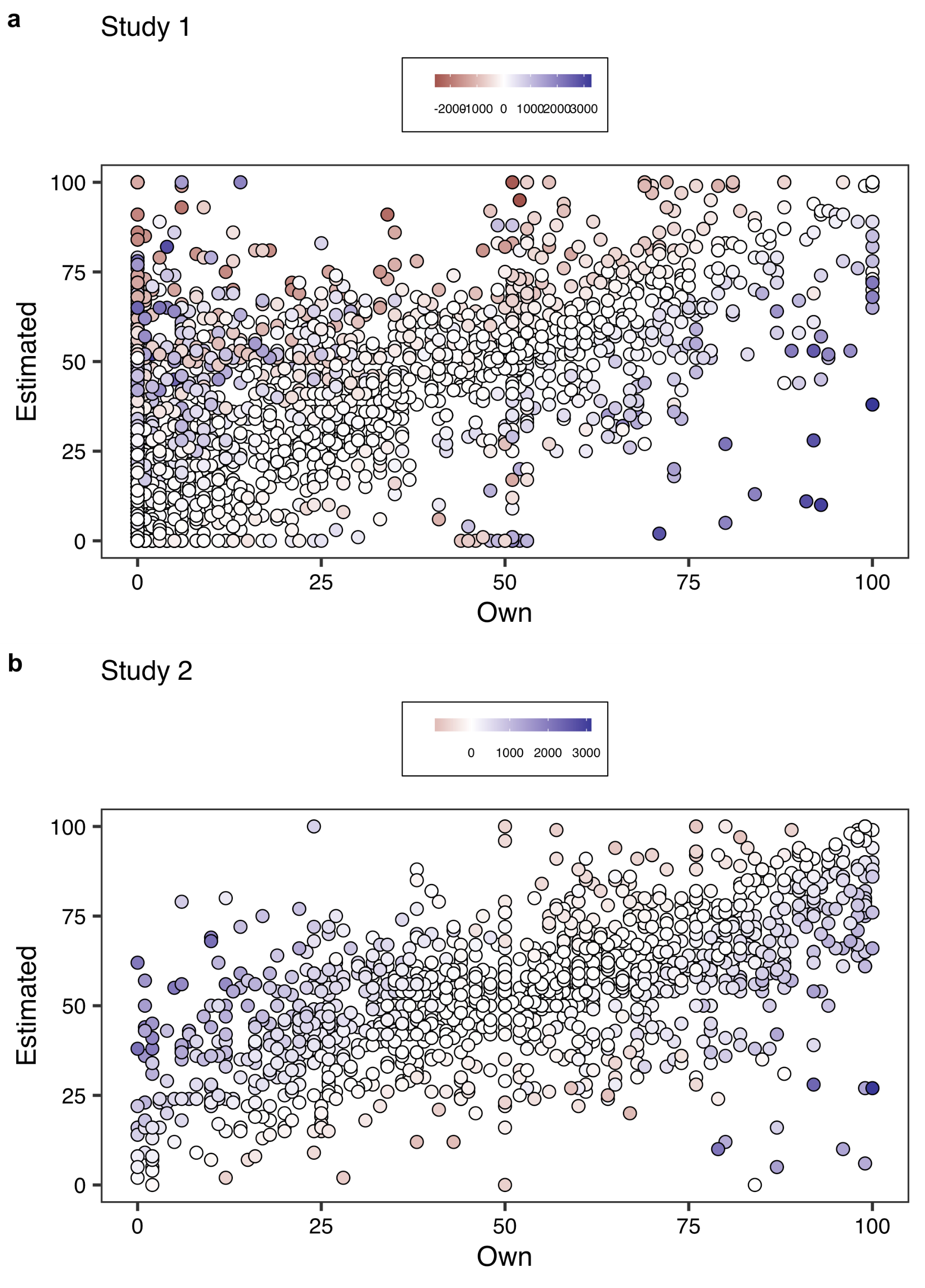


Fig. S7. Visualizations for rating values of opinions (X and Y axis) and the improvement by using our method (colour coding). The bluish colour indicates that our method improved opinions and the reddish colour that our method worsened opinions. The whitish colour indicates our method neither improved nor worsened opinions (i.e., the improvement was around 0).

**S9. Detailed results of our data**


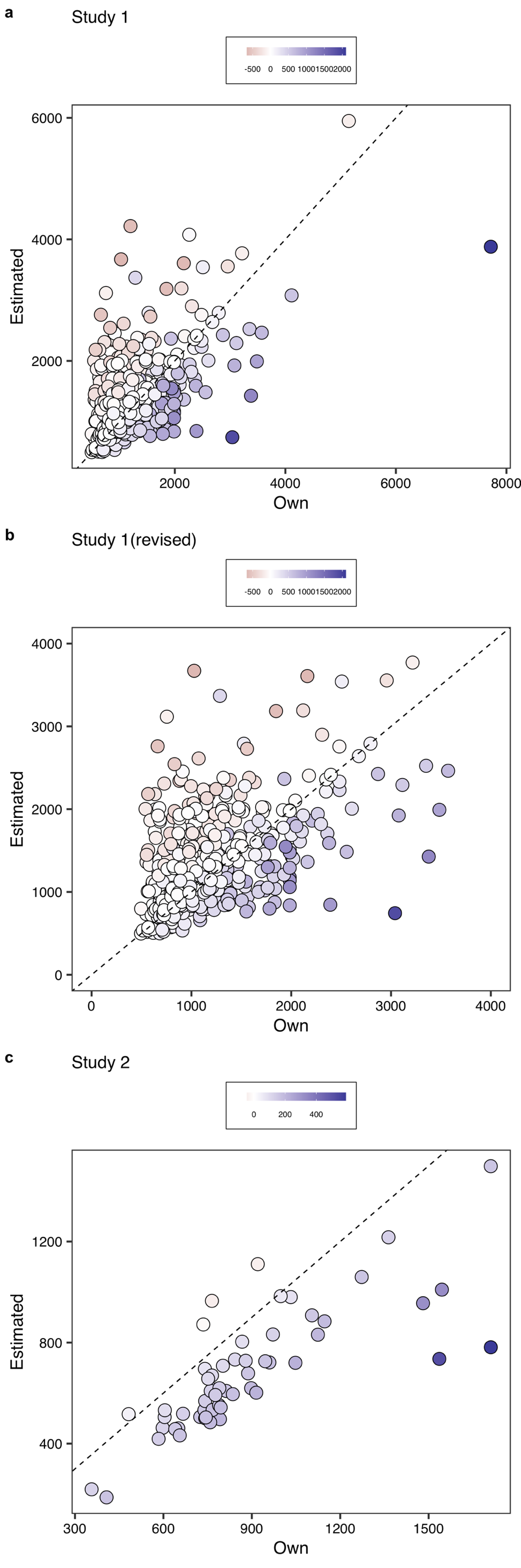

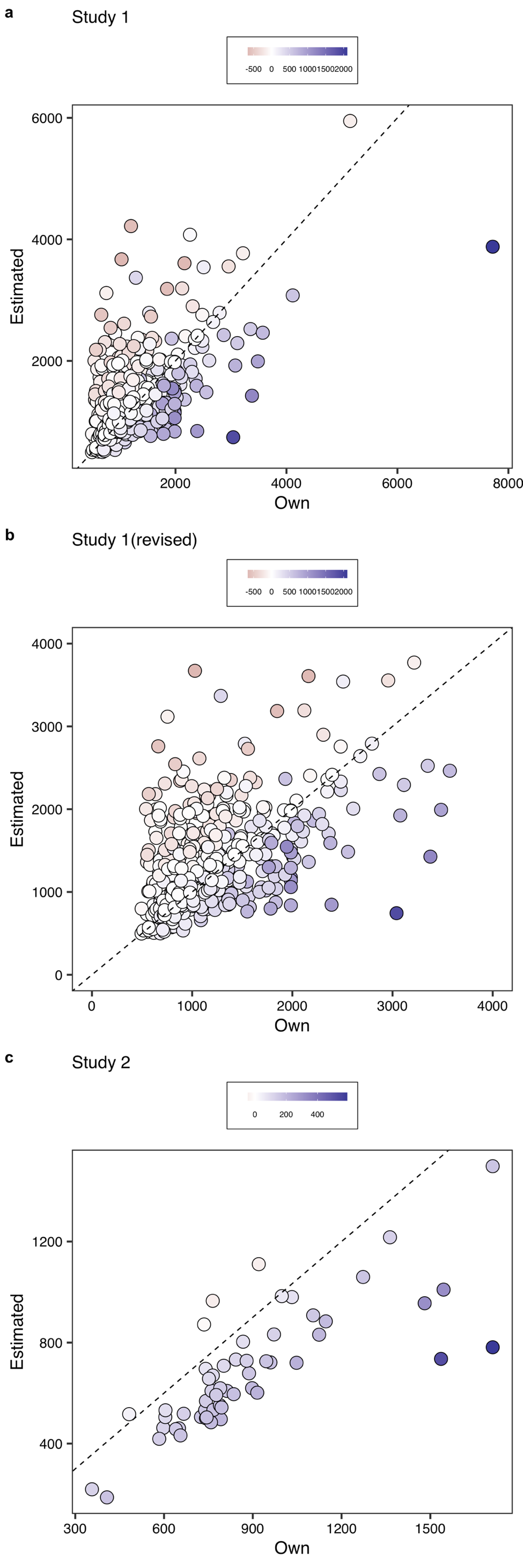
In this section, we adopted a colour coding strategy, similar to that used in Analytis et al.^3,4^)—in particular, MSE of Own (Guessed) opinion on the horizontal (vertical) axis and then colour code error reduction.


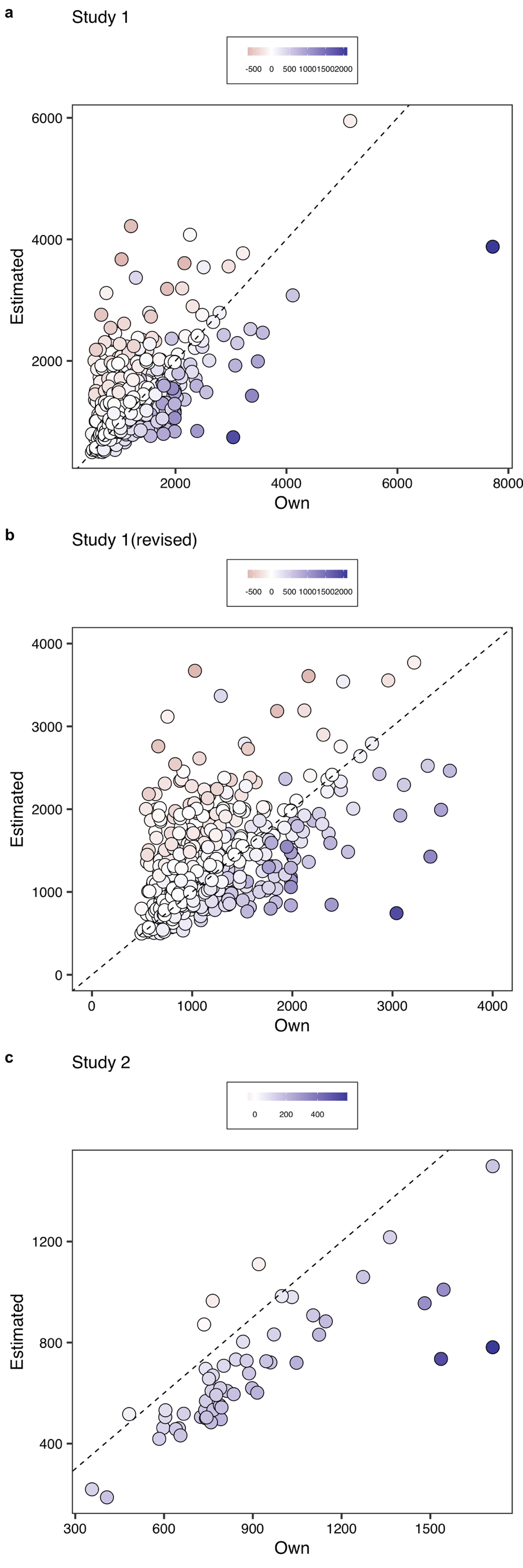


Fig. S8. The detailed results of the data. The bluish colour indicates that the efficacy of our method was high, while the lighter colour shows that the efficacy of our method was low. The reddish colour indicates our method worsened the opinions. For simplicity, Fig. S8b shows the case where the X and Y value was from 0 to 4,000 in Study 1.

Table S4. The experimental stimuli used in Study 1

|  | Condition 1 | Condition 2 | Condition 3 | Condition 4 | Condition 5 |
| --- | --- | --- | --- | --- | --- |
| Painting 1 | Kahlo, *Two Fridas.* | Garsia, *Apocalypse of Saint-Server.* | Buoninsegna, *Virgin and Child Enthrone.* | Dewing, *The Piano.* | Pissaro, *Landscape with Flooded Fields.* |
| Painting 2 | Eakins, *The Gross Clinic.* | Heda, *Still Life with Oysters, Rum Glass, and Silver Cup.* | Brueghel, *Netherlandish Proverbs.* | Vermeer, *The Letter.* | Matisse, *Seated Riffian.* |
| Painting 3 | de Kooning, *Woman.* | Dali, *Gala and Tigers.* | Picasso, *Reclining Nude.* | Matisse, *The Blue Room.* | Cassatt, *Self Portrait.* |
| Painting 4 | Pollock, *Number One.* | Rothko, *Red and Orange.* | Newman, *Eve.* | Hopper, *The Gas Station.* | Hokusai-Katsushika, *Thirty-six Views of Mt. Fuji.* |
| Painting 5 | Van Eyck, *Man in a Turban.* | Henri, *Laughing Child.* | Holbein, *Portrait of Dirk Tybis.* | Cezanne, *Still Life with Kettle.* | Cassatt, *On the Balcony During Carnival.* |

Table S5. The experimental stimuli used in Study 2

| Artist*, Album* | Set 1 | Set 2 |
| --- | --- | --- |
| Antônio Carlos Jobim, *Wave* | Wave (1) | The Red Blouse (13) |
| Berliner Philharmonisches Orchester: Herbert von Karajan, *Mozart Divertimento No. 17* | Divertimento No. 17 in D major K. 334 (2) | Serenata Notturna D-dur, K. 239 (14) |
| Bill Evans, *Portrait in Jazz* | Come Rain or Come Shine (3) | Autumn Leaves (15) |
| Bob Marley, *100% Bob Marley Hits* | I’m Going Home (4) | I Made a Mistake (16) |
| Earth, Wind & Fire, *Greatest Hits* | September (5) | Boogie Wonderland (17) |
| Eminem, *The Eminem Show* | White America (6) | Business (18) |
| Enya, *Amarantine* | Amarantine (7) | It’s in the Rain (19) |
| Guns N’ Roses, *Appetite for Destruction* | It’s So Easy (8) | Nightrain (20) |
| Ikimonogakari, *My song Your song* | Kimagure Romantic (9) | Blue Bird (21) |
| Neil Young, *After the Gold Rush* | Tell Me Why (10) | After the Gold Rush (22) |
| Oasis, *(What’s the Story) Morning Glory?* | Don’t Look Back in Anger (11) | Roll with It (23) |
| Yellow Magic Orchestra, *Public Pressure* | Rydeen (12) | Solid State Survivor (24) |


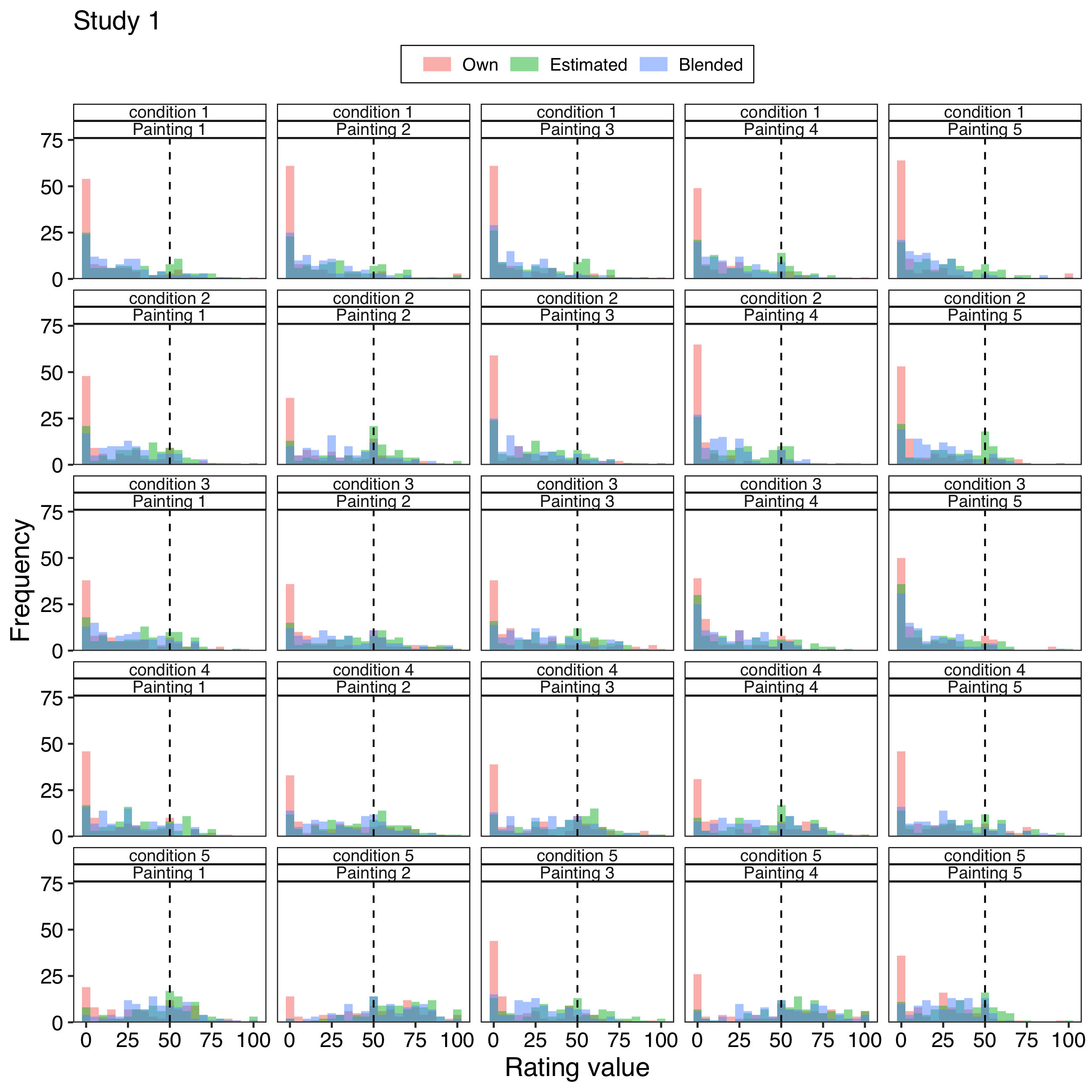


Fig. S9. Rating value on each Painting in Study 1. Specifically, in Own opinion, a number of rating values were zero


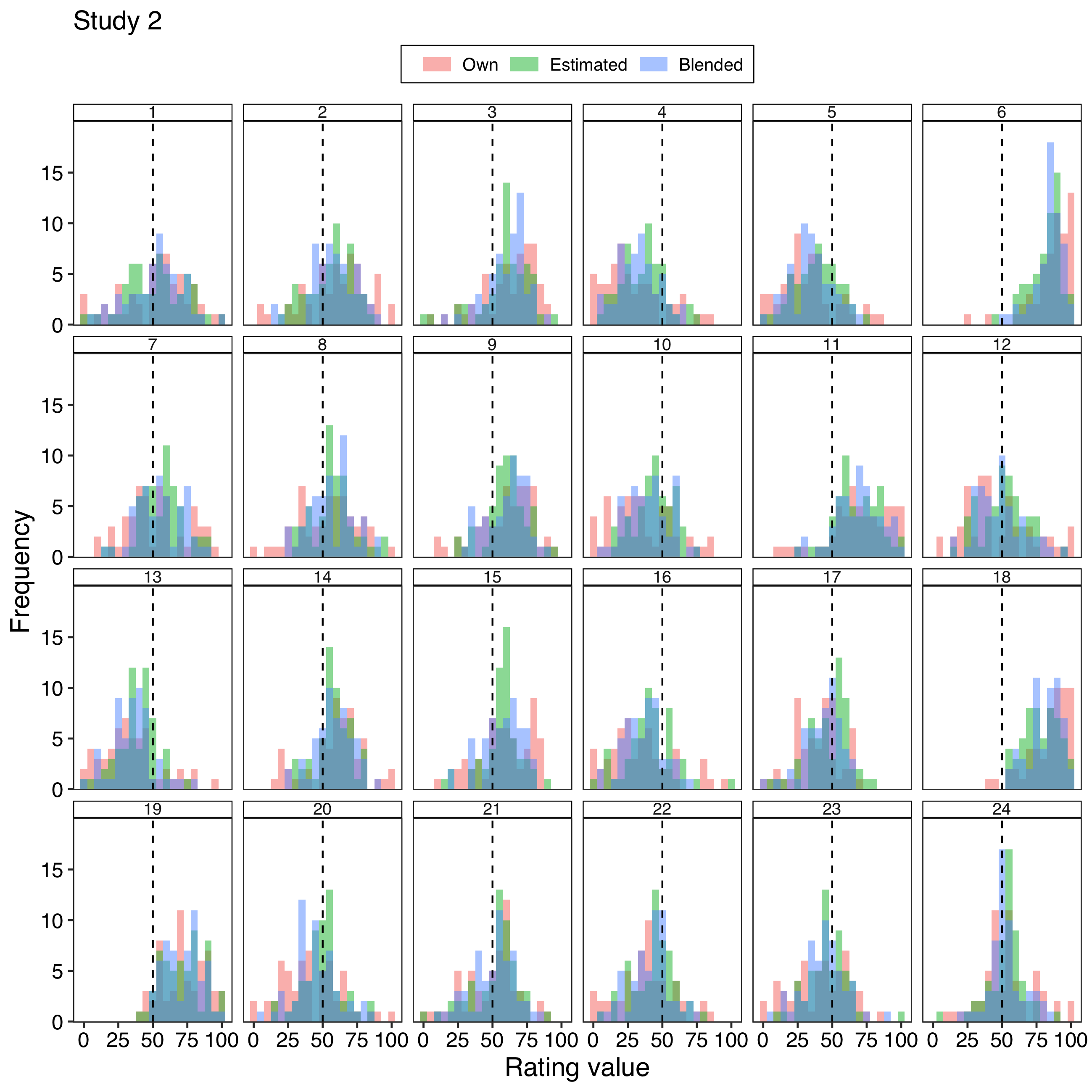


Fig. S10. The rating value on each music piece in Study 2. The rating values tended to follow normal distribution across all types of opinions.

1. Yaniv, I., Choshen-Hillel, S. & Milyavsky, M. Receiving advice on matters of taste: Similarity, majority influence, and taste discrimination. *Organ. Behav. Hum. Decis. Process.* **115**, 111–120 (2011).

2. Müller-Trede, J., Choshen-Hillel, S., Barneron, M. & Yaniv, I. The Wisdom of Crowds in Matters of Taste. *Manage. Sci.* **64**, 1779-1803.

3. Analytis, P. P., Barkoczi, D. & Herzog, S. M. Social learning strategies for matters of taste. *Nat. Hum. Behav.* **2**, 415–424 (2018).

4. Analytis, P. P., Barkoczi, D., Lorenz-Spreen, P. & Herzog, S. The Structure of Social Influence in Recommender Networks. *Proc. Web Conf.* 2655–2661 (2020).
